# Self-rated health is associated with sleep quality in teacher education students

**DOI:** 10.3389/fpubh.2026.1879385

**Published:** 2026-06-26

**Authors:** Ivana Nikolić, Tomislav Hublin, Marko Badrić

**Affiliations:** 1 Department of Kinesiology, Faculty of Teacher Education, University of Zagreb, Zagreb, Croatia; 2 Department of Social Studies, Međimurje University of Applied Sciences in Čakovec, Čakovec, Croatia

**Keywords:** cardiorespiratory fitness, health behaviors, self-rated health, sleep quality, teacher education students, university students

## Abstract

**Background:**

Sleep problems are common among university students and are associated with lifestyle behaviors, health status, and overall wellbeing. Examining factors associated with sleep quality in teacher education students may provide valuable insights into student wellbeing and sleep-related health risks.

**Objective:**

This study examined whether health behaviors, self-rated health, and objectively measured cardiorespiratory fitness predict sleep quality among teacher education students.

**Methods:**

A total of 228 students completed the Pittsburgh Sleep Quality Index (PSQI) and a questionnaire assessing alcohol consumption, cigarette and energy drink use, body mass index, and self-rated health. Cardiorespiratory fitness was assessed using the 20-metre shuttle run test. Participants were classified as good or poor sleepers using the standard PSQI cut-off (>5). Hierarchical logistic regression was conducted in three steps: Model 1 included age, sex, and relationship status, Model 2 added health and health-related variables, and Model 3 included VO₂max.

**Results:**

Poor sleep quality was reported by 36.4% of students. In hierarchical logistic regression analysis, self-rated health was the only significant predictor of poor sleep quality. Compared with students reporting very good or excellent health, those reporting good health (OR = 2.91; 95% CI: 1.54–5.47; *p* < 0.001) and poor/very poor health (OR = 3.16; 95% CI: 1.02–9.76; *p* = 0.046) had higher odds of poor sleep quality. Cardiorespiratory fitness (VO₂max) was not independently associated with sleep quality in the final regression model. The final model demonstrated modest explanatory power (Nagelkerke *R*^2^ = 0.120).

**Conclusion:**

Self-rated health showed the most consistent association with sleep quality among teacher education students, suggesting that students’ perceptions of their overall health may serve as a simple indicator of wellbeing in this population.

## 1 Introduction

Quality sleep plays a crucial role in maintaining mental and physical health, particularly during adolescence and young adulthood, when students are exposed to considerable academic and emotional demands (1, 2). During this developmental period, sleep regulation is influenced by behavioral, physiological, and psychosocial factors. From a socioecological perspective, everyday health behaviors such as alcohol consumption, smoking, energy drink consumption and insufficient physical activity may disrupt sleep–wake rhythms and contribute to poorer sleep quality (3–5). Physiological characteristics, including cardiorespiratory fitness and body mass index, reflect broader aspects of health and may influence sleep through mechanisms related to metabolic regulation and overall physiological functioning (6–9). Psychosocial stressors are also highly relevant in university settings, where irregular schedules, academic pressure and emotional strain are frequently associated with impaired sleep continuity and lower subjective sleep quality. Self-rated health integrates these behavioral, physiological and emotional influences, making it a sensitive indicator of general wellbeing (10, 11).

Epidemiological studies indicate that inadequate sleep is common among university students, with between 37.6 and 75.6% exceeding the Pittsburgh Sleep Quality Index (PSQI) cut-off for poor sleep quality. For example, 48.7% of students in Germany (12), 53.7% in China (13) and 75.6% in Brazil (4) reported poor sleep quality, while 37.6% of Croatian students were classified as poor sleepers according to the same criterion (14). A bibliometric analysis has also documented a marked increase in research on sleep among students over the past decade, reflecting growing recognition of sleep as an important determinant of student wellbeing (15).

Physical activity and cardiorespiratory fitness have attracted increasing interest in relation to sleep regulation. Cardiorespiratory fitness, commonly assessed through maximal oxygen uptake (VO₂max), is widely recognized as an objective indicator of aerobic capacity and one of the strongest predictors of long-term health and mortality (6). Because cardiorespiratory fitness reflects long-term physiological adaptation to habitual physical activity, it may also be relevant for understanding sleep-related physiological processes. Meta-analytic evidence suggests that physical activity may improve several aspects of sleep, including sleep duration, latency and subjective sleep quality (16, 17). A recent meta-analysis in adult women reported that structured exercise interventions were associated with reductions in PSQI scores, indicating improvements in subjective sleep quality (18). Similarly, a network meta-analysis among adults with sleep disorders found that moderate aerobic exercise and mind–body exercise modalities were among the most effective non-pharmacological approaches for improving sleep outcomes (19). These findings highlight the potential role of physical activity as a behavioral strategy for improving sleep, although the magnitude of the effect may depend on exercise type, intensity and timing (20).

However, despite growing research on sleep among university students, relatively little is known about the combined influence of health behaviors and objectively assessed cardiorespiratory fitness on sleep quality among students enrolled in predominantly female teacher education programs. Previous studies have often examined lifestyle behaviors, physical activity or psychosocial factors separately, while fewer studies have investigated their combined contribution to sleep quality within specific student groups.

The aim of this study was to examine the extent to which health behaviors (smoking, alcohol consumption and energy drink intake), cardiorespiratory fitness (VO₂max), body mass index, subjective health and sociodemographic characteristics predict sleep quality in a population of students enrolled in teacher education and preschool education study programs. Based on previous research, it was hypothesized that poorer health behaviors and lower cardiorespiratory fitness would be associated with higher odds of poor sleep quality.

## 2 Methods

### 2.1 Research procedures and sample of participants

Of the 244 students initially included, 16 were excluded from the final analysis due to incomplete questionnaires, chronic illnesses or injuries that prevented participation in the 20-m shuttle run test, or lack of informed consent. The final sample consisted of 228 students enrolled in teacher and preschool education programs at the Faculty of Teacher Education, University of Zagreb, with a mean age of 21.96 ± 4.24 years. The sample was predominantly female (94.7%; n = 216), reflecting the gender distribution typical of education-related study programs and professions in Croatia. National labor statistics indicate that women account for approximately 79% of employees in the Croatian education sector (21).

The study was conducted in December 2025 during regular Physical Education classes. As part of their course activities, students completed standard assessments, including the 20-m shuttle run test and anthropometric measurements (height and weight). Following the testing session, students were invited to voluntarily complete an anonymous questionnaire via a Google Forms link. Participation was anonymous and voluntary, and only adult students who provided informed consent were included in the study. The study was approved by the Ethics Committee of the Faculty of Teacher Education, University of Zagreb (protocol code 641–03/25–01/12; approval date 26 November 2025). All procedures were conducted in accordance with the ethical standards of the institutional research committee and the Declaration of Helsinki.

### 2.2 Measurement of sleep quality

Sleep quality was assessed using the Pittsburgh Sleep Quality Index (PSQI), a validated instrument that measures subjective sleep quality over the past month (22). The questionnaire consists of 19 self-rated items grouped into seven components: subjective sleep quality, sleep latency, sleep duration, habitual sleep efficiency, sleep disturbances, use of sleep medication, and daytime dysfunction. Each component is scored from 0 to 3, and the global PSQI score ranges from 0 to 21, with higher scores indicating poorer sleep quality. In accordance with the original PSQI recommendations, a global PSQI score greater than 5 indicates poor sleep quality, while scores of 5 or lower indicate good sleep quality (22). This threshold has also been supported in psychometric evaluations of the PSQI in college student populations (23). Based on this criterion, sleep quality was dichotomized (good vs. poor) and used as the dependent variable in subsequent analyses.

### 2.3 Anthropometric measurements

BMI was calculated as body weight in kilograms divided by height in meters squared (kg/m^2^). Body height and body weight were measured using standardized anthropometric procedures, with participants wearing light clothing and no shoes. Body weight was measured using a Tanita RD-545 bioelectrical impedance analyzer (Tanita Corporation, Tokyo, Japan) to the nearest 0.1 kg, while body height was measured using an anthropometer to the nearest 0.1 cm. BMI categories were defined according to the World Health Organization criteria (24): underweight (BMI < 18.5), normal weight (18.5–24.9), overweight (25.0–29.9), and obesity (≥ 30.0). For logistic regression analysis, BMI was dichotomized into two categories: (1) normal weight (including underweight) and (2) overweight/obesity.

### 2.4 Measurement of cardiorespiratory fitness

Maximal oxygen uptake (VO₂max) was estimated using the 20-m multistage shuttle run test as originally described by Léger and Lambert (25). Participants ran back and forth between two lines placed 20 meters apart while following standardized audio signals that dictated the running pace. The test commenced at 8.0 km/h and increased by 0.5 km/h at each stage. The test was terminated when participants reached volitional exhaustion or failed to reach the line in time on two consecutive occasions. The speed of the last completed stage was used to estimate VO₂max according to the regression equation reported by Léger and Lambert (25): VO₂max (mL·kg^−1^·min^−1^) = 5.857 × speed (km/h) − 19.458.

### 2.5 Health-related variables

Self-rated health was assessed using a single-item question: “In general, how would you rate your health?,” with responses on a five-point Likert scale ranging from 1 (very poor) to 5 (excellent). This single-item measure has been widely validated and shown to predict important health outcomes, including mortality, chronic diseases, and sleep quality (10, 11, 26). For the purposes of logistic regression analysis, responses were grouped into three categories: very poor/poor (1–2), good (3), and very good/excellent (4–5).

Consumption of cigarettes, alcohol and energy drinks was assessed using a frequency scale and categorized into three groups: (1) never, (2) occasionally (at least once a month but not weekly), and (3) often (at least once a week or almost every day). For regression analyses, responses were dichotomized into No (never) and Yes (occasional or frequent consumption).

### 2.6 Relationship characteristics

Relationship status was assessed using a question about current partnership status. For the purposes of logistic regression analysis, the variable was coded as a binary variable (0 = not in a relationship, 1 = in a relationship).

### 2.7 Statistical analyses

Data were analyzed using IBM SPSS Statistics version 29. Descriptive statistics, including means, standard deviations, and percentages, were calculated for all variables. Chi-square tests were used to examine differences in categorical variables between students with good and poor sleep quality. Independent samples *t*-tests were used for continuous variables (age and VO₂max). All statistical tests were two-tailed, and statistical significance was set at *p* < 0.05.

Associations between the continuous PSQI score and predictor variables were explored using Spearman correlations as a preliminary descriptive analysis. Logistic regression was then used as the primary multivariable analysis with dichotomized sleep quality as the outcome variable. Prior to logistic regression, multicollinearity among predictors was assessed using variance inflation factors (VIF). All VIF values were below 2, indicating no evidence of multicollinearity. The assumption of linearity in the logit for the continuous predictor (VO₂max) was assessed using the Box–Tidwell procedure. The test did not indicate a statistically significant deviation from linearity (*p* = 0.086).

Hierarchical binary logistic regression was used to examine predictors of poor sleep quality. Logistic regression was chosen to facilitate interpretation of predictors of clinically relevant poor sleep based on the established PSQI threshold (>5). Predictors were entered into the model in three consecutive steps. Model 1 included sociodemographic variables (age, sex, and relationship status). Model 2 added health and health-related variables: self-rated health (dummy-coded with “very good/excellent” as the reference category), BMI (normal weight/underweight vs. overweight/obesity), alcohol consumption (No vs. Yes), cigarette use (No vs. Yes), and energy drink consumption (No vs. Yes). Model 3 included all variables from Model 2 and added VO₂max as a continuous indicator of cardiorespiratory fitness. This hierarchical approach allowed examination of the incremental contribution of each block of predictors and changes in explained variance across models. Two sensitivity analyses were conducted to examine the robustness of the findings. First, the hierarchical logistic regression models were repeated using a stricter definition of poor sleep quality (PSQI ≥ 7). Second, a modified PSQI score excluding the subjective sleep quality and daytime dysfunction components was calculated to reduce potential conceptual overlap with self-rated health. Because the modified PSQI score was analyzed as a continuous outcome, associations with the modified score were examined using multiple linear regression analysis including the same predictors as in the primary regression models.

## 3 Results

Table 1 presents the descriptive characteristics of the sample and differences between students with good and poor sleep quality. The two groups did not differ significantly in age, sex, VO₂max, relationship status, BMI, alcohol consumption, cigarette use, or energy drink consumption (all *p* > 0.05). The only significant difference was observed for self-rated health (χ^2^ = 15.13, *p* < 0.001), with better perceived health more common among students reporting good sleep quality.

**Table 1:** Sociodemographic and health characteristics of the participants.

Variable	Categories	Good sleep (*n* = 145)	Poor sleep (*n* = 83)	Test statistic (*p*-value)
Age (M ± SD)		21.74 ± 3.84	22.35 ± 4.86	*t*(226) = −1.037; *p* = 0.301
Sex	Male	7 (4.8%)	5 (6.0%)	χ^2^(1) = 0.15; *p* = 0.697
	Female	138 (95.2%)	78 (94.0%)	
Relationship status	Not in a relationship	52 (35.9%)	35 (42.2%)	χ^2^(1) = 0.89; *p* = 0.346
	In a relationship	93 (64.1%)	48 (57.8%)	
BMI	Normal weight/underweight	97 (66.9%)	52 (62.7%)	χ^2^(1) = 0.42; *p* = 0.517
	Overweight/obesity	48 (33.1%)	31 (37.3%)	
Self-rated health	Poor/very poor	7 (4.8%)	8 (9.6%)	**χ** ^**2**^ **(2) = 15.13; *p* < 0.001**
	Good	34 (23.4%)	37 (44.6%)	
	Very good/excellent	104 (71.7%)	38 (45.8%)	
Alcohol consumption	No	38 (26.2%)	23 (27.7%)	χ^2^(1) = 0.06; *p* = 0.805
	Yes	107 (73.8%)	60 (72.3%)	
Cigarette use	No	93 (64.1%)	45 (54.2%)	χ^2^(1) = 2.18; *p* = 0.140
	Yes	52 (35.9%)	38 (45.8%)	
Energy drink consumption	No	104 (71.7%)	54 (65.1%)	χ^2^(1) = 1.10; *p* = 0.294
	Yes	41 (28.3%)	29 (34.9%)	
VO₂max (mL·kg^−1^·min^−1^), M ± SD		36.79 ± 3.89	37.07 ± 3.62	*t*(226) = −0.53; *p* = 0.597

Data are presented as mean ± standard deviation or *n* (%). Percentages for categorical variables are presented within sleep-quality groups. Poor sleep quality was defined as PSQI > 5. Variables are presented in the order of inclusion in the regression models. Independent samples *t*-tests were used for age and VO₂max, whereas chi-square tests were used for categorical variables. Bold values indicate statistically significant differences (*p* < 0.05).

Spearman’s correlation analysis (Table 2) showed a significant negative correlation between the total PSQI score and self-rated health (*ρ* = −0.306, *p* < 0.001), indicating that students who perceived their health more favorably also reported better sleep quality. Energy drink consumption showed a weak positive correlation with PSQI score (ρ = 0.129, *p* = 0.051), although the association was not statistically significant. Other variables, including VO₂max, body mass index, alcohol consumption, and cigarette use, were not significantly associated with PSQI score.

**Table 2:** Correlation between the PSQI score and predictor variables.

Variable	Spearman’s rho	*p*
VO₂max	0.026	0.693
BMI	0.018	0.783
Self-rated health	−0.306	**<0.001**
Alcohol consumption	0.108	0.103
Cigarette use	0.049	0.465
Energy drink consumption	0.129	0.051

Higher PSQI scores indicate poorer sleep quality. Bold values indicate statistically significant correlations (*p* < 0.05).

The complete results of the three hierarchical logistic regression models predicting poor sleep quality are presented in Table 3. In Model 1, age, sex, and relationship status were not significantly associated with sleep quality. Model 2 added health-related variables, including self-rated health, BMI, alcohol consumption, cigarette use, and energy drink consumption. In this model, self-rated health was the only variable significantly associated with poor sleep quality. Compared with students reporting very good or excellent health, those reporting good health had nearly threefold higher odds of poor sleep quality (OR = 2.825; 95% CI: 1.510–5.284; *p* = 0.001). None of the other health-related variables were significantly associated with sleep quality. In Model 3, the addition of cardiorespiratory fitness (VO₂max) did not meaningfully change the results. VO₂max was not significantly associated with poor sleep quality (OR = 1.059; 95% CI: 0.975–1.150; *p* = 0.173). Self-rated health remained the only significant predictor in the final model. Compared with students reporting very good or excellent health, those reporting good health (OR = 2.905; 95% CI: 1.543–5.471; *p* < 0.001) and those reporting poor/very poor health (OR = 3.158; 95% CI: 1.022–9.760; *p* = 0.046) had higher odds of poor sleep quality.

**Table 3:** Logistic regression analysis of predictors of poor sleep quality among students (*n* = 228).

Predictor	Model 1 OR (95% CI)	*p*	Model 2 OR (95% CI)	*p*	Model 3 OR (95% CI)	*p*
Age	1.040 (0.976–1.108)	0.227	1.042 (0.969–1.120)	0.270	1.046 (0.972–1.126)	0.228
Sex (female vs. male)	0.920 (0.272–3.113)	0.894	0.975 (0.262–3.635)	0.970	1.243 (0.317–4.885)	0.755
Relationship status (in a relationship)	0.728 (0.408–1.297)	0.281	0.742 (0.403–1.368)	0.339	0.718 (0.388–1.328)	0.291
Self-rated health	—	—	Wald χ^2^(2) = 11.728	0.003	Wald χ^2^(2) = 12.457	0.002
Poor/very poor vs. very good/excellent	—	—	2.751 (0.910–8.321)	0.073	3.158 (1.022–9.760)	0.046
Good vs. very good/excellent	—	—	2.825 (1.510–5.284)	0.001	2.905 (1.543–5.471)	<0.001
BMI (overweight/obesity vs. normal weight/underweight)	—	—	0.828 (0.440–1.557)	0.558	0.925 (0.481–1.779)	0.815
Alcohol consumption (Yes vs. No)	—	—	0.752 (0.386–1.466)	0.403	0.757 (0.387–1.478)	0.414
Cigarette use (Yes vs. No)	—	—	1.506 (0.825–2.751)	0.183	1.589 (0.863–2.925)	0.137
Energy drink consumption (Yes vs. No)	—	—	1.360 (0.719–2.571)	0.344	1.329 (0.700–2.523)	0.384
VO₂max (per 1 mL·kg^−1^·min^−1^ increase)	—	—	—	—	1.059 (0.975–1.150)	0.173

Reference categories: very good/excellent self-rated health, No alcohol consumption, No cigarette use, No energy drink consumption, normal weight/underweight, not in a relationship, and male sex. Alcohol consumption, cigarette use, and energy drink consumption were coded as binary variables (Yes vs No). Model 1 included age, sex, and relationship status; Model 2 additionally included self-rated health, BMI, alcohol consumption, cigarette use, and energy drink consumption; Model 3 additionally included VO₂max. Model fit statistics: Model 1, Nagelkerke *R*^2^ = 0.014, Hosmer–Lemeshow *p* = 0.570; Model 2, Nagelkerke *R*^2^ = 0.110, Hosmer–Lemeshow *p* = 0.531; Model 3, Nagelkerke *R*^2^ = 0.120, Hosmer–Lemeshow *p* = 0.381, Omnibus χ^2^(10) = 20.969, *p* = 0.021.

A sensitivity analysis using a stricter PSQI cut-off (≥7) showed a similar pattern of results, with self-rated health remaining the strongest predictor of poor sleep quality. Under this stricter definition, energy drink consumption was also associated with higher odds of poor sleep quality (Supplementary Table S1). A second sensitivity analysis using a modified PSQI score excluding the subjective sleep quality and daytime dysfunction components showed that self-rated health remained significantly associated with the modified PSQI score, whereas the other predictors were not significant (Supplementary Table S2).

## 4 Discussion

This study examined the association between health behaviors, cardiorespiratory fitness and sleep quality among teacher education students. Poor sleep quality (PSQI >5) was reported by 36.4% of students, which is similar to the prevalence reported among Croatian university students (37.6%) (14), and slightly lower than international estimates, where the prevalence of poor sleepers typically ranges between 45 and 76% (4, 13, 27). These differences may reflect cultural and academic factors, as well as the timing of data collection. In the present study, data were collected outside examination periods, which may have reduced acute stress levels and the prevalence of sleep disturbances.

Self-rated health emerged as the only significant predictor of sleep quality and the factor most consistently associated with poor sleep outcomes across all analyses. Students who rated their health less favorably were more likely to report poor sleep quality, with the strongest difference observed between those who perceived their health as very good or excellent and those with lower health ratings. This finding is consistent with previous research indicating that self-rated health is a broad and multidimensional indicator of physical and psychological functioning that reflects individuals’ overall perceptions of their health status (10, 11). Similar associations between poorer perceived health and reduced sleep quality have also been reported among university students (12).

The observed association may partly reflect the multidimensional nature of self-rated health, which incorporates physical health, emotional functioning, stress, fatigue, and health-related behaviors. As a global assessment of health, it may capture aspects of subjective wellbeing that are not fully reflected by objective health indicators alone. In the context of sleep, self-rated health may partly reflect symptoms such as fatigue, reduced energy, impaired daily functioning, and diminished wellbeing, all of which are closely related to sleep experiences. Consequently, some degree of conceptual overlap between self-rated health and subjective sleep measures may be expected. Research has shown that academic stress may negatively affect sleep onset and maintenance among university students (1), while poor sleep quality has also been associated with higher perceived stress and lower physical and mental health-related quality of life in this population (28). In addition, chronic sleep disruption has been associated with adverse metabolic and broader health outcomes (9). These mechanisms may help explain the association between self-rated health and sleep quality observed in the present study.

In university students, self-rated health may be particularly informative because it provides a simple yet comprehensive indicator of overall wellbeing. This may be especially relevant during higher education, a period characterized by academic demands, lifestyle changes, and psychosocial challenges that can influence both health perceptions and sleep-related behaviors. Given the close relationship observed between perceived health and sleep quality, health promotion initiatives that support healthy lifestyle behaviors and effective stress management may contribute to improvements in both sleep quality and perceived health.

In the final regression model, both students reporting good health and those reporting poor or very poor health had higher odds of poor sleep quality compared with students reporting very good or excellent health. The confidence interval for the poor or very poor health category was relatively wide, likely reflecting the small number of participants in this group. The overall explanatory power of the regression model was modest (Nagelkerke *R*^2^ = 0.120), which is common in studies examining behavioral and psychosocial determinants of sleep. The cross-sectional design also precludes conclusions regarding the direction of the observed association, as poorer sleep quality may itself contribute to less favorable perceptions of overall health. The association between self-rated health and sleep quality remained significant in both sensitivity analyses, including the stricter PSQI cut-off and the modified PSQI score excluding the subjective sleep quality and daytime dysfunction components. These findings strengthen confidence in the robustness of the observed association and suggest that it was not solely dependent on the definition of poor sleep quality or on potential conceptual overlap between self-rated health and specific PSQI components.

Cardiorespiratory fitness, measured through VO₂max and analyzed as a continuous variable, was not associated with sleep quality in the present study. The estimated odds ratio corresponds to approximately 1.24 per one standard deviation increase in VO₂max (≈3.8 mL·kg^−1^·min^−1^), although this association remained statistically non-significant. This finding contrasts with a previous study reporting that higher levels of cardiorespiratory fitness were associated with better sleep quality among adolescent girls (29). However, evidence linking cardiorespiratory fitness and subjective sleep quality remains inconsistent across adolescent and young adult populations. Similarly, with the present findings, Osailan et al. (8) reported no association between objectively measured VO₂peak and PSQI scores in apparently healthy adults, suggesting that aerobic fitness alone may not be a reliable predictor of subjective sleep quality in this age group.

Several factors may help explain the absence of a significant association between cardiorespiratory fitness and sleep quality in the present study. First, the sample was relatively homogeneous, consisting predominantly of young women enrolled in similar teacher education programs, which may have limited variability in both fitness levels and health-related behaviors. In addition, cardiorespiratory fitness reflects long-term physiological adaptation to habitual physical activity (6), whereas sleep patterns in student populations may be more strongly influenced by short-term behavioral and psychosocial factors such as stress, daily routines, and evening habits (1, 27). Furthermore, the PSQI is a subjective measure of perceived sleep quality, whereas VO₂max reflects physiological fitness capacity. These measures capture different dimensions of health and may therefore not necessarily show strong associations within relatively healthy young adult populations. The inconsistent findings reported across studies may also reflect differences in study populations, fitness assessment methods, and sleep-related measures. Taken together, these factors may partly explain the absence of a significant relationship between cardiorespiratory fitness and subjective sleep quality observed in the present study.

Because of the cross-sectional design, the present study could not determine the direction of the observed associations or capture potential dynamic and bidirectional relationships between cardiorespiratory fitness and subjective sleep quality over time. Another potential explanation relates to physiological changes associated with the menstrual cycle, which may influence both VO₂max performance and perceived sleep quality. Menstrual-cycle-related fluctuations in thermoregulation, circadian rhythms, and sleep patterns have been well documented (30, 31) and may obscure associations between fitness and sleep in predominantly female samples. These factors were not assessed in the present study and should be considered in future research. Longitudinal studies incorporating objective sleep measures, physical activity assessment, and menstrual cycle characteristics may provide a more comprehensive understanding of the relationship between cardiorespiratory fitness and sleep quality in young adults.

Health-related behaviors such as alcohol consumption, cigarette smoking and energy drink use were not independently associated with sleep quality in the main regression model when other variables were taken into account. Although previous studies have reported associations between these behaviors and poorer sleep outcomes in student populations (32–34), their impact often depends on dose, timing and long-term patterns of use. In the present study, levels of consumption may have been relatively low or too infrequent to produce measurable effects. It is also possible that the influence of these behaviors was attenuated after adjustment for other health-related variables included in the model.

A sensitivity analysis using a stricter PSQI cut-off (≥7) produced a broadly similar pattern of results, with self-rated health remaining the most consistent predictor of poor sleep quality. In addition, energy drink consumption emerged as a significant predictor under the stricter definition of poor sleep quality, suggesting that stimulant-related behaviors may be more strongly associated with more severe sleep disturbances. However, the relatively small number of participants classified as poor sleepers under the stricter cut-off should be considered when interpreting these findings.

Relationship or marital status has been identified as a significant correlate of sleep quality in some university student populations (35). Likewise, BMI was not identified as a significant predictor of sleep quality, consistent with previous research in university students (36).

This study has several limitations. Sleep quality was assessed using the PSQI, which relies on subjective reporting and may be influenced by recall bias. In addition, both self-rated health and sleep quality were assessed using self-reported measures. Therefore, shared-method variance cannot be completely excluded and may have contributed to the observed association between these variables. The cross-sectional design prevents conclusions about causality. The sample consisted predominantly of women, which limits generalizability and did not allow sex-specific analyses. In addition, hormonal fluctuations across the menstrual cycle may have influenced both VO₂max performance and perceived sleep quality. Finally, the relatively narrow range of cardiorespiratory fitness values and the lack of data on habitual physical activity patterns and menstrual cycle phases may have limited interpretation of the VO₂max findings.

Despite these limitations, the study provides insight into factors associated with sleep quality among predominantly female teacher education students. The findings suggest that students’ perceptions of their general health may be more strongly associated with sleep quality than specific health behaviors or cardiorespiratory fitness. These results point to the value of wellbeing-oriented university initiatives, particularly those addressing stress management, emotional health and recovery, as strategies for supporting sleep quality in this population.

## 5 Practical implications

These results have several practical implications for supporting wellbeing in teacher education students, a population that is predominantly female. The consistent association between self-rated health and sleep quality suggests that a simple self-rated health question may serve as an accessible screening indicator for identifying students who may be experiencing reduced wellbeing or early signs of sleep problems. Including such brief screening tools within routine student services or wellbeing programs may help recognize potential difficulties at an early stage.

Cardiorespiratory fitness (VO₂max) was not associated with sleep quality in this sample. Nevertheless, the findings suggest that approaches addressing stress management, recovery habits and emotional wellbeing may be particularly relevant for supporting sleep quality in this population. This does not diminish the importance of physical activity for general health but highlights the value of balanced strategies that support both recovery and psychological wellbeing.

Developing awareness of early signs of reduced wellbeing, including changes in perceived health and sleep patterns, may help support student health and wellbeing during higher education. These implications should be interpreted with caution given the cross-sectional design and relatively homogeneous sample.

## 6 Conclusion

Self-rated health was the strongest predictor of sleep quality among teacher education students, whereas other health-related behaviors and cardiorespiratory fitness (VO₂max) were not significantly associated with sleep quality. Students’ perceptions of their general health may therefore serve as a simple indicator of wellbeing in this population. Understanding factors associated with sleep quality during teacher education may help inform efforts to support student wellbeing and reduce sleep-related health risks in higher education settings.

## Data Availability

The raw data supporting the conclusions of this article will be made available by the authors, without undue reservation.
