# Supplementary material for: Self-rated health is associated with sleep quality in teacher education students
Source: Front Public Health. 2026 Jun 26;14:1879385. doi: 10.3389/fpubh.2026.1879385 (PMC13350231; doi:10.3389/fpubh.2026.1879385)
Supplement: Supplementary file 1 [file Table_1.DOCX]

Supplementary Material

# Supplementary Tables

*Supplementary Table S1* *Sensitivity analysis using a stricter PSQI cut-off (≥7) for poor sleep quality*

| Variable | OR | 95% CI | p |
| --- | --- | --- | --- |
| Age | 1.011 | 0.929–1.101 | .795 |
| Sex (female vs male) | 1.194 | 0.244–5.857 | .827 |
| Relationship status (in a relationship) | 0.779 | 0.384–1.578 | .488 |
| Self-rated health overall | Wald χ²(2) = 18.061 | — | <.001 |
| Poor/very poor vs very good/excellent | 5.344 | 1.575–18.128 | **.007** |
| Good vs very good/excellent | 4.292 | 2.082–8.849 | **<.001** |
| BMI (overweight/obesity vs normal weight) | 0.491 | 0.223–1.080 | .077 |
| Alcohol consumption (Yes vs No) | 0.763 | 0.348–1.672 | .499 |
| Cigarette use (Yes vs No) | 1.787 | 0.888–3.596 | .103 |
| Energy drink consumption (Yes vs No) | 2.325 | 1.140–4.744 | **.020** |
| VO₂max (per 1 mL·kg⁻¹·min⁻¹ increase) | 1.002 | 0.910–1.103 | .971 |

***Note.*** Poor sleep quality was defined as PSQI ≥7 (good sleep: 0–6). Odds ratios were obtained from multivariable logistic regression analysis. Reference categories: self-rated health = very good/excellent; energy drink consumption = No; cigarette use = No; alcohol consumption = No; BMI = normal weight/underweight; relationship status = not in a relationship; sex = male. Alcohol consumption, cigarette use, and energy drink consumption were dichotomised into No vs Yes categories for regression analyses. Model fit statistics: Nagelkerke R² = .196; Hosmer–Lemeshow p = .912; Omnibus χ²(10) = 32.116, p < .001.

*Supplementary Table S2 Sensitivity analysis using a modified PSQI score excluding subjective sleep quality and daytime dysfunction components*

| Variable | B | SE | β | t | p |
| --- | --- | --- | --- | --- | --- |
| Sex | -0.049 | 0.155 | -0.022 | -0.317 | .751 |
| Age | 0.012 | 0.008 | 0.104 | 1.472 | .143 |
| Relationship status | -0.061 | 0.069 | -0.060 | -0.885 | .377 |
| BMI | -0.052 | 0.073 | -0.050 | -0.709 | .479 |
| Self-rated health | -0.220 | 0.054 | -0.274 | -4.049 | <.001 |
| Alcohol consumption | 0.032 | 0.075 | 0.029 | 0.427 | .669 |
| Cigarette use | 0.011 | 0.069 | 0.011 | 0.160 | .873 |
| Energy drink consumption | 0.020 | 0.073 | 0.019 | 0.272 | .786 |
| VO₂max | 0.002 | 0.009 | 0.017 | 0.240 | .810 |

*Note.* The dependent variable was the continuous modified PSQI score calculated after excluding the subjective sleep quality and daytime dysfunction components. Alcohol consumption, cigarette use, and energy drink consumption were coded as binary variables (No vs Yes). Model fit: R = .304, R² = .092, adjusted R² = .055.
